# Abundant and Diverse RNA Viruses in Insects Revealed by RNA-Seq Analysis: Ecological and Evolutionary Implications

**DOI:** 10.1128/mSystems.00039-20

**Published:** 2020-07-07

**Authors:** Haoming Wu, Rui Pang, Tong Cheng, Liang Xue, Haiyan Zeng, Tao Lei, Moutong Chen, Shi Wu, Yu Ding, Jumei Zhang, Mang Shi, Qingping Wu

**Affiliations:** a State Key Laboratory of Applied Microbiology Southern China, Guangdong Provincial Key Laboratory of Microbiology Culture Collection and Application, Guangdong Open Laboratory of Applied Microbiology, Guangdong Institute of Microbiology, Guangdong Academy of Sciences, Guangzhou, China; b Department of Food Science and Technology, Jinan University, Guangzhou, China; c School of Medicine, Sun Yat-sen University, Guangzhou, China; d School of Life and Environmental Sciences and Sydney Medical School, The University of Sydney, New South Wales, Australia; Princeton University

**Keywords:** insect, RNA virus, virome, virus evolution, ecology

## Abstract

Increasing data indicate that insects serve as major reservoirs and vectors of viruses, which account for the continuously increasing ecological burden and infectious disease outbreaks. Uncovering the hidden diversity of viruses in insects will further the understanding of the ecological and evolutionary perspectives in the emergence of insect-associated virus diseases. In this study, we queried transcriptome sequencing (RNA-Seq) data from more than 600 species across 32 insect orders dwelling in different ecological habitats and recovered more than 1,213 RNA viruses that were recapitulated in 40 families, 2 unclassified genera, and many unspecified viral groups. These novel viruses included the well-known insect-associated viruses within *Flaviviridae*, *Picornavirales*, *Bunyavirales*, *Mononegavirales*, *Nidovirales*, *Reoviridae*, and *Negevirus*. More appeared to form novel clusters within previously described taxa or could be resolved as paraphyletic, including the first astrovirus identified in insects, in which many were sufficiently divergent to warrant the establishment of new virus genera or families. Additionally, some viruses were closely related to the recognized plant-, fungus-, and vertebrate-specific species, implying the importance of relationships between insect behavior and virus spread. Comparative genome analyses also revealed high genomic variability with respect to the flexible gene pool and genome architecture of these newly described viruses, including the evidence for genome reshuffling first discovered in *Dicistroviridae*. The data reflecting the genetically and ecologically diverse viral populations in insects greatly expand our understanding of RNA viruses in nature and highlight that the biodiversity of RNA viruses remains largely unexplored.

**IMPORTANCE** Insects comprise the largest proportion of animals on earth and are frequently implicated in the transmission of vector-borne diseases. However, considerable attention has been paid to the phytophagous and hematophagous insects, with results that provide insufficient and biased information about the viruses in insects. Here, we have delivered compelling evidence for the exceptional abundance and genetic diversity of RNA viruses in a wide range of insects. Novel viruses were found to cover major categories of RNA viruses, and many formed novel clusters divergent from the previously described taxa, dramatically broadening the range of known RNA viruses in insects. These newly characterized RNA viruses exhibited high levels of genomic plasticity in genome size, open reading frame (ORF) number, intergenic structure, and gene rearrangement and segmentation. This work provides comprehensive insight into the origin, spread, and evolution of RNA viruses. Of course, a large-scale virome project involving more organisms would provide more-detailed information about the virus infections in insects.

## Abstract (precis)

Insects comprise the largest proportion of animals on earth and are frequently implicated in the transmission of vector-borne diseases. However, considerable attention has been paid to the phytophagous and hematophagous insects, with results that provide insufficient and biased information about the viruses in insects. Here, we have delivered compelling evidence for the exceptional abundance and genetic diversity of RNA viruses in a wide range of insects. Novel viruses were found to cover major categories of RNA viruses, and many formed novel clusters divergent from the previously described taxa, dramatically broadening the range of known RNA viruses in insects. These newly characterized RNA viruses exhibited high levels of genomic plasticity in genome size, open reading frame (ORF) number, intergenic structure, and gene rearrangement and segmentation. This work provides comprehensive insight into the origin, spread, and evolution of RNA viruses. Of course, a large-scale virome project involving more organisms would provide more-detailed information about the virus infections in insects.

## INTRODUCTION

Viruses are ubiquitous on earth and are deemed to infect all known cellular organisms across the global ecosystems (1, 2). Historically, virus identification has focused on specific organismal communities, such as humans and clinically and commercially associated animals and plants. However, these represent only a small proportion of the range of viral biodiversity in nature and, to a great extent, the data have led to biased inference in characterizing the evolutionary landscape of viruses. An enormous amount of viruses remains undiscovered using classical viral detection methods due to the inability to cultivate the vast majority of viruses *in vitro*. The application of viral metagenomics has facilitated the identification of unknown viruses in a broad host range (3), from unicellular organisms to mammals, terrestrial or aquatic (4–8). Because of their extreme abundance and adaptability in diverse environments and habitats, animals have been implicated frequently in the evolution and spread of viruses. Recent studies have described abundant and diverse RNA viruses in animals, providing important insights about the host adaptation and distribution of viruses (9–14). Still, the hidden diversity of RNA viruses within the ecologically and geographically diverse animal species remains to be explored.

Insects are arguably the most widespread and diverse group of animals on the planet, occupying different ecological niches and playing a dominant role in the functioning of ecosystems (15). Viral infections have been invoked as a significant threat to many ecologically and commercially important insects, with dramatic declines seen in the species richness and diversity in the agricultural and forest ecosystems (16, 17). For the plant sap- and blood-sucking insects, the food and feeding habits make them hot spots for interspecies transmission and emergence of novel viruses (18–20). Indeed, more than 70% of the known plant viruses depend on insect vectors for their survival and spread (21). In the past decades, the increased incidence of insect-borne epidemics has been documented, including the new emergence of epidemic Zika virus and reemergence of dengue (DENV), chikungunya (CHIKV), yellow fever (YFV), and other infectious diseases (22, 23). These viruses are transmitted between their hosts by the insect vectors, causing no overt pathology during circulation in insect populations (24). Though many more viruses have been persistently identified in insects, little is known about their ecological distribution and evolutionary dynamic in the wild.

Over time, ecological species identification has revealed large and diverse populations of insects, as well as of their parasites (25). As obligate cellular parasites, viruses share intimate relationships with their hosts, raising issues concerning the genetic landscape and macroevolutionary patterns of the fast-evolving RNA viruses in insects. Previous studies focused on those insects with public health and economic importance or on those restricted to specific habitats, such as mosquitoes, honey bees, and flies (13, 26–31). Few studies have been performed on the insects as a whole. As a result of the deluge of omics studies, an unprecedented explosion in biological data (i.e., genomics and transcriptomics) is providing a particularly abundant resource for virus discovery (5, 8, 32). Here, we conducted a systematic study to explore RNA viruses of more than 600 insect species by querying publicly available transcriptome sequencing (RNA-Seq) data. Our findings revealed the underlying RNA virus communities in a wide range of insects, representing a comprehensive study investigating the unexplored RNA viruses in insects, as well as their evolutionary and ecological implications.

## RESULTS

### Abundant and divergent RNA viruses identified in insects.

We performed the virus discovery analyses on a curated collection of RNA-Seq data available publicly in databases. The data set contained 664 species spanning 32 orders of insects (see Data Set S1 in the supplemental material). The assembled contigs were subjected to BLAST analyses against the reference viral databases. This resulted in ∼16,990 virus-associated contigs from 648 insect species, of which 89.35% were associated with two or more virus species (Data Set S1). To elucidate the evolutionary aspect of the newly identified viruses, we extracted the putative viral contigs that had significant matches to the highly conserved viral RNA-dependent RNA polymerase (RdRp) domain and reconstructed the phylogenetic trees together with the recognized taxa of RNA viruses proposed by the International Committee on Taxonomy of Viruses (ICTV [https://talk.ictvonline.org/taxonomy/vmr/]), as well as some unclassified RNA viruses occupying important phylogenetic positions. A total of 1,213 contigs representing complete or partial viral genomes were recovered from the RNA-Seq data for 484 insect species (484/664, 72.89%) (see Fig. S1a in the supplemental material; see also Data Set S1). Phylogenetic analysis demonstrated that these putative RNA viruses fell within or in close proximity to a wide range of recognized viral taxa, including 40 viral families and 2 unclassified genera, as well as newly described RNA virus groups, such as *Qinviridae* and *Chuviridae* (Data Set S1). Nevertheless, BLAST analysis and pairwise comparisons revealed that the sequences of most of the newly identified RNA viruses were highly divergent from the previous reported viral sequences: almost 1,194 (91.01%) shared less than 70% amino acid (aa) identity with the most closely related viruses, while 471 (35.93%) shared less than 40% (Data Set S1; see also Fig. S1b).

10.1128/mSystems.00039-20.1 FIG S1 (a) Box plot representing the size distribution of the identified RdRp-encoding contigs by virus clade. (b) Histogram plot showing the distribution of amino acid sequence identities between the identified RdRp-encoding contigs and the best-matching viral sequences. Download FIG S1, EPS file, 1.6 MB. Copyright © 2020 Wu et al. 2020 Wu et al. This content is distributed under the terms of the Creative Commons Attribution 4.0 International license.

10.1128/mSystems.00039-20.8 DATA SET S1 Summary of the data set used and the RNA viruses identified in this study. Download Data Set S1, XLSX file, 2.1 MB. Copyright © 2020 Wu et al. 2020 Wu et al. This content is distributed under the terms of the Creative Commons Attribution 4.0 International license.

**FIG 1:**
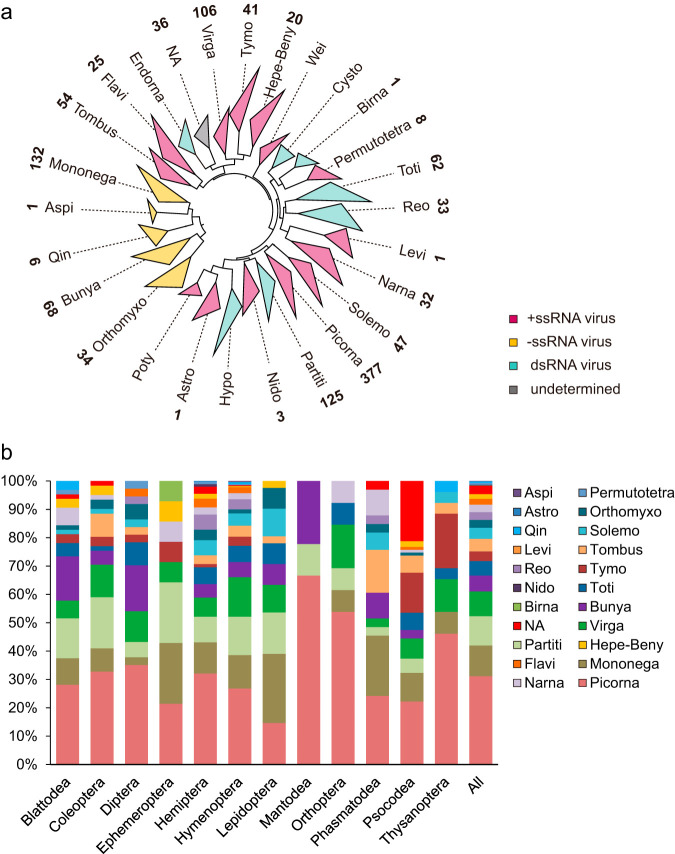
Taxonomic diversity and distribution of RNA viruses in insects. (a) Taxonomic reassessment of the RNA viruses based on RdRp. For each phylogeny, branches are collapsed and are displayed as triangles filled with different colors (see legend). The name of each clade is abbreviated based on the collapsed viral families or orders. The number of viral contigs assigned to each clade is indicated by the Arabic numerals next to the clade name. (b) Percent relative abundance of RNA viruses across each insect order. Those insect orders with too few samples (<10 samples) analyzed in our data set are not listed individually. Astro, *Astroviridae*; Aspi, *Aspiviridae*; Hepe-Beny, *Benyviridae*, *Hepeviridae*, *Alphatetraviridae*, *Togaviridae*, *Bastrovirus*; Birna, *Birnavirdae*; Bunya, *Bunyavirales*, *Arenaviridae*; Cysto, *Cystoviridae*; *Endorna*, *Endornaviridae*; Flavi, *Flaviviridae*; *Hypo*, *Hypoviridae*; Levi, *Leviviridae*; Mononega, *Mononegavirales*, *Jingchuvirales*; Narna, *Narnaviridae*, *Botourmiaviridae*; Nido, *Nidovirales*; Partiti, *Partitiviridae*, *Amalgaviridae*, *Picobirnaviridae*; Orthomyxo, *Orthomyxoviridae*; Permutotetra, *Permutotetraviridae*; Poty, *Potyviridae*; Picorna, *Picornavirales*, *Solinviviridaes*, *Caliciviridae*, *Marnaviridae*; Qin, *Qinviridae*; Solemo, *Solemoviridae*, *Luteoviridae*, *Barnaviridae*, *Alvernaviridae*; Reo, *Reoviridae*; *Tombus*, *Tombusviridae*, *Nodaviridae*, *Sinaivirus*, *Carmotetraviridae*, *Luteoviridae*; Toti, *Totiviridae*, *Chrysoviridae*, *Megabirnaviridae*, *Quadriviridae*, *Botybirnavirus*; Tymo, *Tymovirales*; Virga, *Virgaviridae*, *Togaviridae*, *Bromoviridae*, *Closteroviridae*, *Idaeovirus*; Wei, *Weivirus*; NA, unclassified.

The current official taxonomy could not recapitulate the extremely diverse RNA virus populations in insects accurately. Indeed, more than 40% of the novel viruses could not be precisely assigned to the current taxa or exhibited low confidence levels. Instead, we reclassified the RNA viruses into 26 clades as described for previous studies (Fig. 1a) (9, 33). These viral clades were abbreviated based on the representative members of each clade (e.g., “Picorna” is an abbreviation representing *Picornavirales*, *Solinviviridae*, and *Caliciviridae*). Although these viruses were highly divergent, their taxonomic status could be deduced based on the panoramic phylogenies. Newly discovered viruses were assigned into 22 viral clades, of which 13 were relatively ubiquitous as they were observed in more than 10 insect orders (Fig. 1b; see also Data Set S1). The others, including one putative RNA phage within the Levi clade, appeared to be infrequent. A clearly separated group which cannot be confidently assigned to any currently recognized taxon was found on the phylogenies (Fig. 1a). The novel clade comprised 36 viruses that were mostly identified in insect orders *Psocodea* (*n* = 21) and *Hemiptera* (*n* = 9). The closest relative of these viruses was *Agaricus bisporus virus 16* (ABV16), a putative four-segmented virus found in mushroom (25.62% to 44.65% amino acid similarity for RdRp segment, Data Set S1) (34). These viruses might represent a novel viral family or order.

**FIG 2:**
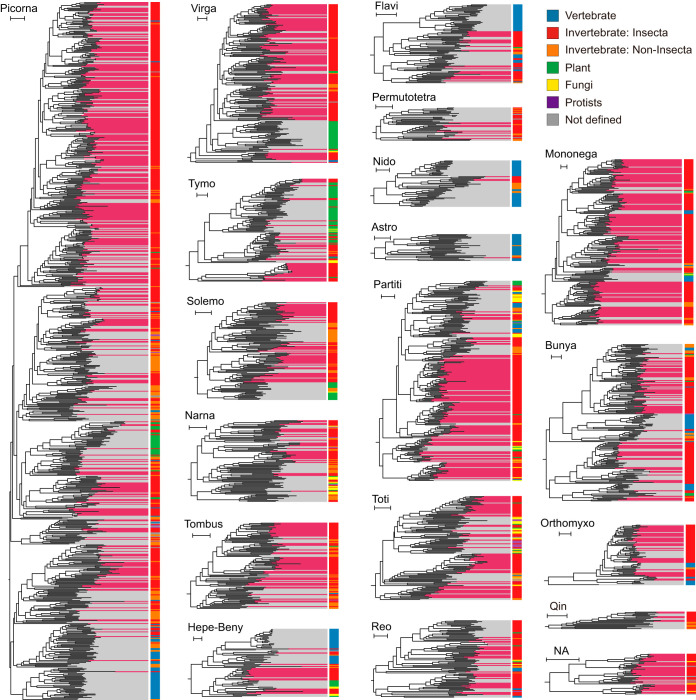
Genetic diversity of RNA viruses in insects. The illustrated phylogenetic trees, which 19 major groups of RNA viruses, were inferred based on RdRp data using the maximum likelihood method and rerooted at midpoint. As indicated, a branch background in red shading represents viruses identified in this study, and a branch background in shading represents those described previously. Host groups are indicated by differently colored bars (see legend). Scale bars represent 0.5 amino acid substitutions per site. Detailed phylogenies are available in Fig. S2 to S6.

The detailed evolutionary landscape was reevaluated by phylogenetic analyses based on all available RNA viruses by clade. As indicated, viruses identified in insects were distributed throughout different phylogenies (Fig. 2; see also Fig. S2 to S6), implying a broader host range and much-more-diverse RNA virus communities in insects. The observed categories of RNA viruses could be comparable to those of the whole invertebrates, although some taxonomic groups appeared to be more highly represented in insects, including the recognized insect-specific iflaviruses, dicistroviruses, bunyaviruses, rhabdoviruses, flaviviruses, and negeviruses. A growing number of novel RNA viruses formed apparent insect-specific lineages within many other clades, such as Hepe-Beny, Orthomyxo, Tombus, Toti, Tymo, Solemo, Narna, Virga, Nido and Partiti (for definitions of the abbreviations of the virus designation that appear here and throughout, see the Fig. 1 legend). It should be noted that closely related species of plant viruses were frequently discovered in insects. These putative plant viruses might be transmitted by insect vectors and were most commonly found in clade Tymo, as well as some new members of *Secoviridae*, *Virgaviridae*, *Partitiviridae*, *Aspiviridae*, *Botourmiaviridae*, and other plant virus species (Data Set S1).

10.1128/mSystems.00039-20.2 FIG S2 Phylogenetic trees of the “Picorna” clade. Viruses identified in this study are labeled by filled circles. Host groups are indicated by different colors (see legend). Branch support values are shown at nodes (SH-aLRT support, >70%; ultrafast bootstrap, >90%). Genome organizations of the represent viruses are plotted and marked with star symbols in the phylogenies. The embedded colored boxes represent conserved protein domains identified by CD-search at NCBI and are denoted by the corresponding labels. Download FIG S2, EPS file, 1.3 MB. Copyright © 2020 Wu et al. 2020 Wu et al. This content is distributed under the terms of the Creative Commons Attribution 4.0 International license.

### Novel positive-sense single-stranded RNA (+ssRNA) viruses.

The picorna-like viruses were significantly overrepresented in insects (Fig. 1b), accounting for up to 31.08% (377/1,213) of the novel viruses, and were present in 33.73% (224/664) of the insect species. Novel picorna-like viruses were found to diversify into multiple different evolutionary lineages (Fig. S2), in which the insect-associated *Iflaviridae* and *Dicistroviridae* were the most abundant (73.7% of all picorna-like viruses). The family *Secoviridae* was the major group of plant picorna-like viruses (35); which six novel strains were identified with 48.3% to 99.0% aa identity to the most closely related plant virus species. Of those, two close relatives of nepoviruses (93.7% nucleotide [nt] pairwise identity) were identified in a pollinating bee (Halictus quadricinctus) and a predatory wasp (Crabro peltarius), respectively, and higher viral relative abundance was identified in Halictus quadricinctus (0.17% total reads). It might be expected that Halictus quadricinctus acted as a transmission vector for nepoviruses. Other plant virus-like sequences were scattered throughout the trees, such as the strains closely related to the plant-infecting Tomato matilda virus (TmaV) within *Iflaviridae*. In addition, some viruses were found to be evolutionarily related to vertebrate-associated species. For example, we observed a new picornavirus in Diplonychus rusticus that best matched Rhimavirus A (39.5% aa identity), a member of the proposed reptile- and amphibian-specific genus *Rafivirus* within the *Picornaviridae* (36). Another virus that was found in Hydrochara caraboides appeared to be closely related to Ampivirus A1 (83.0% aa identity), previously identified in a smooth newt (37). In the unclassified posavirus-like cluster, four novel members were found to be related to the isolates from humans, pigs, rats, and bats, though the identities of the natural hosts have not yet been clarified.

Twenty-five novel flavi-like viruses were identified, including nine in the newly described segmented *Jingmenvirus* (Fig. S3) (38). Novel jingmenviruses clustered together with Wuhan cricket virus (WHCV) and not with the tickborne Alongshan virus (ALSV) and *Toxocara canis* larva agent (TCLA). Notably, a novel jingmenvirus species was present in *Ctenocephalides felis* (cat flea), which might be implicated as a biological vector of Jingmenvirus, as previously described for blood-sucking ticks and mosquitoes (38–40). Another three viruses were clustered with Wenzhou shark flavivirus, representing a disparate evolutionary lineage from *Flavivirus* and *Jingmenvirus*. More viruses fell outside the Flavi-Jingmenvirus, Hepa-Pegivirus, and Pestivirus lineages. These viruses represented a highly divergent subset of unclassified viruses within the Flavi clade.

10.1128/mSystems.00039-20.3 FIG S3 Phylogenetic trees of major viral clades of the positive-sense single-stranded RNA (+ssRNA) viruses (nonpicorna). Viruses identified in this study are labeled by filled circles. Host groups are indicated by different colors (see legend). Branch support values are shown at nodes (SH-aLRT support, >70%; ultrafast bootstrap, >90%). Genome organizations of the represented viruses are plotted and marked with star symbols in the phylogenies. The embedded colored boxes represent conserved protein domains identified by CD-search at NCBI and are denoted by the corresponding labels. Download FIG S3, EPS file, 1.5 MB. Copyright © 2020 Wu et al. 2020 Wu et al. This content is distributed under the terms of the Creative Commons Attribution 4.0 International license.

The newly identified tymovirus-like viruses comprised five major well-supported groups (Fig. S3), among which four groups were affiliated with known plant/fungus virus groups in the order *Tymovirales*. A total of 17 novel strains were recognized as plant viruses. These viruses demonstrated a close relationship with the well-known plant-infecting species, either with a high percentage of identity (70% nt identity with 90% coverage, Data Set S1) or resolved into a single monophyletic clade in the phylogenies. In addition, novel members within the *Maculavirus* were largely hosted by phytophagous or omnivorous insects, representing potential plant viruses (41). Fourteen novel viruses formed a deeply divergent branch distantly related to *Deltaflexiviridae* and were most frequently identified in insect order *Psocodea* (11/14, 78.57%). A BLASTX search revealed that the conserved RdRp domain shared low (26.58% to 34.36%) amino acid similarity with the most closely related viral sequences. The topologies of the phylogenetic trees demonstrated that these viruses belonged to a novel taxonomic group associated with insects, though the host range should be reconfirmed with more data. Within other plant/fungus-associated clades, such as Virga, Solemo, Narna, Tombus, and Hepe-Beny, extremely diverse RNA virus communities were also discovered (Fig. S3). In addition to the putative plant/fungus-infecting strains, most newly identified viruses formed genetically distinct evolutionary lineages, many of which are as yet unclassified. For example, the virgavirus/negevirus-like viruses represented a diverse group of viruses that were associated with insects and other invertebrate animals.

Insect-specific evolutionary lineages were also found in vertebrate-associated virus clades, such as the Nido, Astro, and Hepe clades (Fig. S3). Novel nidoviruses fell into two distinct insect-specific lineages, in which two strains belonged to the previously described *Mesoniviridae*. Another nido-like virus formed a well-supported monophyletic group with Wuhan insect virus 19 and Wuhan nido-like virus 1, representing an unclassified taxonomic group within *Nidovirales*. One distant relative of astrovirus was discovered in a terrestrial insect, Unaspis euonymi (*Hemiptera*: *Diaspididae*). The Unaspis euonymi astro-like virus clustered with two invertebrate-associated astroviruses (host: *Myriapoda* and *Hirudinea*) and fell in a position basal to the astroviruses of vertebrate on the phylogeny, possibly representing ancient divergence of astroviruses from invertebrates. To our knowledge, the Unaspis euonymi astro-like virus was the first astrovirus species identified in insect. One novel bastrovirus strain was also identified in a filter-feeding insect, *Ephemera* sp. *HW-2014*, and shared a moderate level of sequence similarity with two strains isolated from bat and sewage (60.14% and 61.53% aa identity, respectively). Other +ssRNA viruses included nine permutotetraviruses and one levi-like RNA phage (Fig. S3), among which the novel levi-like virus might be associated with the gut microbiota of the host Lepismachilis y-signata.

### Novel negative-sense single-stranded RNA (−ssRNA) viruses.

The mononega-like viruses were the most common −ssRNA viruses identified, presenting in more than 15% insects in this study. Altogether, newly identified mononega-like viruses diversified into nine major evolutionary groups, including six in *Mononegavirales* (Fig. S4). We observed two clearly separate groups within the *Rhabdoviridae*, of which one was related to plant-associated *Nucleorhabdovirus* and the other belonged to vertebrate-associated *Almendravirus*. It may be hypothesized that insect-associated rhabdoviruses arose independently. Other mononegaviruses included members within the *Lispiviridae*, *Xinmoviridae*, and *Artoviridae* lineages and a Borna-like lineage. Thirty-five viruses were assigned to the newly established *Chuviridae* lineage. These chuviruses were divided into three groups with huge differences in their genome architectures, indicating a possible relationship between the genome arrangement and diversity.

10.1128/mSystems.00039-20.4 FIG S4 Phylogenetic trees of major viral clades of the negative-sense single-stranded RNA (−ssRNA) viruses. Viruses identified in this study are labeled by filled circles. Host groups are indicated by different colors (see legend). Branch support values are shown at nodes (SH-aLRT support, >70%; ultrafast bootstrap, >90%). Genome organizations of the represented viruses are plotted and marked with star symbols in the phylogenies. The embedded colored boxes represent conserved protein domains identified by CD-search at NCBI and are denoted by the corresponding labels. Download FIG S4, EPS file, 1.4 MB. Copyright © 2020 Wu et al. 2020 Wu et al. This content is distributed under the terms of the Creative Commons Attribution 4.0 International license.

Sixty-nine bunya-like viruses were identified, of which most could be classified into three insect-specific groups, namely, *Goukovirus*, *Orthophasmavirus*, and an unclassified sister group to *Phenuiviridae* (Fig. S4). Within the *Feravirus*, two nearly identical strains (>99% nt identity) were identified in Trialeurodes vaporariorum and its parasitic counterpart Encarsia formosa, respectively, providing evidence of its importance with respect to feeding behavior in virus transmission. A novel tenuivirus strain was identified in Archaeopsylla erinacei and was found to be closely related to the Rice stripe tenuivirus (53.8% aa identity). Another strain obtained from a phytophagous insect, Larinus minutus, was distantly related to the plant-infecting Citrus concave gum-associated virus. These bunya-like viruses, including the arena-like virus identified in *Heteropsilopus ingenuus*, were assumed to be trisegmented, though the data have shown that some viral genomes lack one or two segments due to inadequate sequencing reads or extremely divergent features.

A remarkable diversity of orthomyxo-like viruses was revealed in insects (Fig. S4). These viruses formed multiple insect-specific lineages in *Quaranjavirus*. In addition, two thogotovirus variants were present in Plea minutissima and shared 51.64% amino acid sequence identity. Consistently, these viruses possessed five to six segments, although some genomic segments were missed and some might be redundant. Other −ssRNA viruses included six strains within the newly described bisegmented *Qinvirus* and one within the plant-infecting *Ophiovirus* (Fig. S4). The aspi-like virus was identified in a phytophagous insect, Panurgus dentipes, and was assigned to a new species in *Ophiovirus* with an unknown natural plant host.

### Novel double-sense double-stranded RNA (dsRNA) viruses.

A genetically diverse population was found in clade Partiti with a broad host range, including novel members within *Partitiviridae* and *Amalgaviridae*. More than 125 partitiviruses formed seven major groups, in which 31 novel strains were assigned to the recognized plant/fungus-infecting *Alphapartitivirus* and *Betapartitivirus* (Fig. S5). However, few were identified in genera *Gammapartitivirus* and *Cryspovirus*. Although a novel clade was identified adjacent to the *Deltapartitivirus*, these viruses were distantly related to the phytopathogenic virus species and might represent an invertebrate-specific lineage. Another four groups cannot be classified into any recognized taxa; one of the group was associated with the plant-infecting Maize-associated partiti-like virus. Members of the family *Partitiviridae* were believed to have two genomic segments. However, the capsid-encoded segment was rarely identified within these novel virus groups. It seemed that a much more diverse parititivirus community existed in insects, which might act as major reservoirs and/or transmission vectors for the parititivirus hosted by plants or fungi. In addition, three nonsegmented parititi-like viruses were found in omnivorous insects Thanasimus formicarius, Tetrodontophora bielanensis, and Haliplus fluviatilis. These novel viruses were phylogenetically related to the plant-infecting species within the *Amalgaviridae* and shared similar genome organizations, with two overlapping open reading frames (ORFs).

10.1128/mSystems.00039-20.5 FIG S5 Phylogenetic trees of major viral clades of the double-sense double-stranded RNA (dsRNA) viruses. Viruses identified in this study are labeled by filled circles. Host groups are indicated by different colors (see legend). Branch support values are shown at nodes (SH-aLRT support, >70%; ultrafast bootstrap, >90%). Genome organizations of the represented viruses are plotted and marked with star symbols in the phylogenies. The embedded colored boxes represent conserved protein domains identified by CD-search at NCBI and are denoted by the corresponding labels. Download FIG S5, EPS file, 0.9 MB. Copyright © 2020 Wu et al. 2020 Wu et al. This content is distributed under the terms of the Creative Commons Attribution 4.0 International license.

Three main virus groups within the Toti clade were found in insects (Fig. S5). These viruses showed limited sequence similarity with the recognized totiviruses. For example, 20 newly identified toti-like viruses clustered together with Hubei toti-like virus 22 and formed an apparently separate lineage distinct from those of fungus/protozoa giardiaviruses. Another group contained one plant virus strain, Persimmon latent virus, and could be regarded as a sister group to those noninsect viruses. However, novel viruses were occasionally found in the genera *Victorivirus* and *Trichomonasvirus* and one unclassified taxon associated with protozoa. Other toti-like viruses included botybirna- and chryso-like viruses. The botybirnavirus was found in a predatory insect, Grylloblatta bifratrilecta, with 30.7% aa identity with the most closely related viruses. The chryso-like viruses were identified in a phytophagous insect, Psyllopsis fraxinicola, and a parasitic wasp, Eurytoma brunniventris, with a typically four-segmented genome corresponding to those of members of the *Chrysoviridae* family.

Thirty-three reo-like viruses were identified, among which 11 strains were novel members of genus *Orbivirus* and 13 were related to the plant-associated *Fijivirus* (Fig. S5). Other strains were affiliated with different evolutionary lineages of the genera *Coltivirus*, *Phytoreovirus*, *Seadornavirus*, and *Oryzavirus* and four unclassified groups. However, none of the novel reo-like viruses was found in the insect-specific genus *Cypovirus*. Among them, two reo-like viruses were found to be identical to the previously reported strains isolated from *Diaphorina citri* and *Nilaparvata lugens*, respectively (>96% nt identity for all segments), implying wide distribution in the wild. However, most reo-like viruses were highly divergent from all known species; in particular, the multisegmented genome organization made them somewhat more variable.

One birnavirus was identified in a filter-feeding insect, Isonychia kiangsinensis. The genome of Isonychia kiangsinensis birna-like virus comprised two segments, of which the polymerase-encoded segment best matched the *Drosophila* X virus (42.8% aa identity). The birnavirus was divided into three evolutionary lineages (Fig. S5); one was associated with vertebrate animals, and the other two were associated with invertebrate animals (mostly insects). Hence, a genetically diverse population of birnaviruses might be present in insects and other invertebrate animals.

### Great genome flexibility in insect viruses.

**FIG 3:**
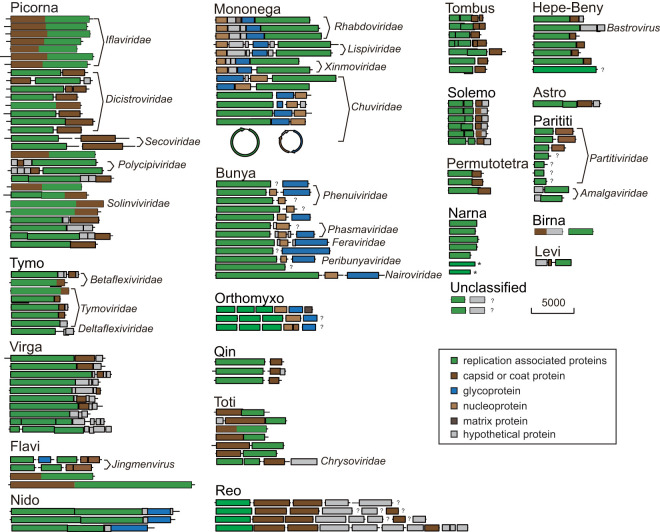
Genome architectures and comparison of representative viruses within major viral clades. The genomes are drawn as boxes and lines approximately to scale, representing open reading frames (ORFs) and noncoding regions, respectively. The predicted homologous genes are shown in colored boxes (see legend). The question marks (?) represent the missing genomic segments, and each asterisk (*) represents a novel hypothetical ORF on the minus strand of the virus genome.

Though the overall levels of sequence similarity were very low even within each virus clade, some genes were conserved among RNA viruses. The most ubiquitous and conserved motifs were associated with the viral polymerase (Fig. S7a). In general, the key conserved residues arrange themselves in an orderly manner and maintain structural integrity in the RNA polymerase domain, with the exception of few viruses (Fig. S7b). Highly conserved motif C (GDD) appeared to be lost in the polymerase active sites of birnaviruses or to be translocated upstream of motifs A and B in permutotetraviruses and negeviruses (A→B→C to C→A→B). Consensus motifs were also discovered in some essential viral proteins, including the RNA helicase, protease, methyltransferase, and coat proteins (Fig. 3). These viral proteins represent the most highly conserved genome components in the majority of RNA viruses, although some of them are alternatively encoded by different viruses.

10.1128/mSystems.00039-20.6 FIG S6 Phylogenetic tree of the unclassified Agaricus bisporus virus 16 (ABV16)-like viruses. Viruses identified in this study are labeled by filled circles. Host groups are indicated by different colors (see legend). Branch support values are shown at nodes (SH-aLRT support, >70%; ultrafast bootstrap, >90%). Genome organizations of the represented viruses are plotted and marked with star symbols in the phylogeny. The embedded colored boxes represent conserved protein domains identified by CD-search at NCBI and are denoted by the corresponding labels. Sequence logos of the key conserved residues in RdRp catalytic motifs A to C are shown at right. Download FIG S6, EPS file, 0.5 MB. Copyright © 2020 Wu et al. 2020 Wu et al. This content is distributed under the terms of the Creative Commons Attribution 4.0 International license.

10.1128/mSystems.00039-20.7 FIG S7 Conserved residues of RNA-dependent RNA polymerase (RdRp). (a) Sequence logos of the key residues in RdRp catalytic motifs A to C. +ssRNA, positive-sense single-stranded RNA; dsRNA, double-stranded RNA; ns −ssRNA, nonsegmented negative-stranded RNA virus; seg −ssRNA, segmented negative-stranded RNA virus. (b) Sequence alignments depicting the internally permuted of RdRp motifs in negeviruses, permutotetraviruses, and birnaviruses. Download FIG S7, EPS file, 2.8 MB. Copyright © 2020 Wu et al. 2020 Wu et al. This content is distributed under the terms of the Creative Commons Attribution 4.0 International license.

Despite the subtle constraints imposed on RNA virus evolution, the insect viruses retained a substantial level of variability in genome size and architectures (Fig. 3; see also Fig. S2 to S6). The narnaviruses had the simplest genome organization of any RNA viruses, yielding to a single open reading frame (ORF) encoding an RNA-dependent RNA polymerase (RdRp). Nevertheless, an additional ORF was found in the complementary strand of the genome for some insect-derived narnaviruses, which encoded a hypothetical protein with unknown function or with no significant homology to known proteins. More differences were identified in virus genomes containing multiple ORFs for nonsegmented viruses or multiple-genome segments for segmented viruses. For example, the virga-like viruses encoded similar panels of nonstructural proteins, while the structural regions were very flexible and characterized by a complex genomic landscape (Fig. 3), which might be associated with a considerable capacity to accommodate different environments and hosts. Within the toti-like viruses, three different genome structures were observed across different evolutionary lineages (Fig. 3), including a single large open reading frame (ORF) or discontinuous ORFs characterized by overlapping or being separated. The difference in gene arrangements might reflect an alternative utilization of translation initiation by toti-like and other related viruses, including ribosomal frameshift by those viruses with overlapped ORFs and termination-reinitiation mechanism by those with separated ORFs.

The viral genomic heterogeneity was also enriched through genome rearrangement and segmentation. In picorna-like viruses, one of the most remarkable genetic differences was the gene modules’ inversion of the structural and nonstructural protein coding regions among different evolutionary groups (Fig. 3). Within the family *Dicistroviridae*, *Ellipsidion* picorna-like virus 2 and related strains were discovered to undergo an event of genome rearrangement, which likely resulted in the formation of a novel evolutionary lineage descended from *Aparavirus* (Fig. 3). Similarly, the unsegmented mononega-like viruses were characterized by the presence of three hallmark genes encoding the nucleoprotein (N), glycoprotein (G), and large polymerase (L) and were distinguished by a pool of hypothetical proteins with no known orthologues (Fig. 3). Nevertheless, the hallmark genes were not conserved in location in the corresponding genomes, characterized by an N-G-L module for *Mononegavirales* and a G-N-L or L-N-G module for *Jingchuvirales*. Although most mononegaviruses were believed to have a monopartite genome, some chuviruses were characterized by a bi- or trisegmented genome, including a putative circular form (Fig. 3). Genome segmentation was also found in flavi- and solemo-like viruses. In most cases, the segmented viruses were quite distinct from the nonsegmented counterparts. Apart from the conserved NS5 and NS3 segments, the gene repertoires possessed by the segmented jingmenviruses are quite different from those of the related nonsegmented flavi-like viruses and formed a well-supported monophyletic group. A major phylogenetic discrepancy was also observed between segmented and nonsegmented solemo-like viruses, although the encoded viral proteins were similar. Alternatively, the genomes of insect-associated beny-like viruses were not segmented as seen with the recognized *Benyvirus*. It seems that the segmented viruses might have originated from the primitive nonsegmented ones. The tight evolutionary link between segmented and nonsegmented viruses might represent the potential mechanisms that make possible the genome shuffling in some RNA viruses, such as the mononega- and picorna-like viruses.

## DISCUSSION

We conducted a systematic investigation of the RNA viruses in insects, in which abundant and highly diversified RNA virus communities were identified. These RNA viruses were dispersed throughout the phylogenetic trees, and many clades were greatly expanded by the presence of the newly identified viruses (Fig. 2), yielding a considerable fraction within the virus biodiversity. In light of the huge insect populations on the planet, it is conceivable that the level of viral diversity in insects is unprecedented and that a large number of viruses still remain unexplored (15). Based on previous studies and our analysis, most insect-associated viruses are phylogenetically related to other invertebrate viruses, characterized by similar evolutionary tempi and modes (Fig. 2) (42). In general, insect-associated viruses exhibit paraphyletic relationships across the phylogenies of the major virus categories and have diversified into distinct taxonomic groups that comprise more than just several well-studied groups such as *Flaviviridae* (43), *Mononegavirales* (44), and *Bunyavirales* (45). The virus communities possessed by insects appear to be much more genetically diverse than those possessed by other taxonomic kingdoms, including plants and the higher vertebrate animals (9, 10, 46). In the absence of adaptive immunity, insects mainly rely on innate responses for pathogen recognition and clearance (47–49). The lack of adaptive immunity increases the level of tolerance of invasive viruses, ensuring viral survival and accelerated adaptation to the host with little or no fitness cost in insects. Higher viral capacity without an adverse pathological response also results in the insects functioning as important incubators and reservoirs for diverse RNA virus populations, facilitating the emergence of coinfection, recombination, cross-species transmission, and novel viruses.

The remarkable diversity of viral populations might indicate high host specificity of viruses in insects, which would support the idea that viruses are evolutionarily specialized to different ecological niches (11, 13, 50). However, the host barriers are not absolute. A significant risk for viral spillover is associated with trophic interactions, including both antagonism (e.g., herbivory, predation, and parasitism) and mutualism (e.g., pollination), in both directions during contact. The observations in our study demonstrate the possibility of interspecies virus transmission in cases in which predator-prey or host-parasitoid encounters occur, including but not limited to insect-insect interactions. For example, the ectoparasitic mite Varroa destructor has facilitated the global spread of deformed wing virus among populations of honeybees, as this method of transmission has allowed circumvention of ecological or evolutionary barriers to infect new hosts (16). Increased direct or indirect contact might confer great opportunities for insect viruses to cross host boundaries. In this respect, the distribution and composition of viruses in insectivorous animals are extensively shaped by insect diet, as indicated by previously reported evidence from bats (13, 51–53). It is commonly thought that hematophagous insects (e.g., mosquitoes, sandflies, and midges) carry a panel of viral pathogens which may be transmitted to humans or animal reservoirs through biting or feeding (54–56). The real risk may be greater than that represented by those examples. Numerous related virus species have been observed in a wide range of insects and can be traced back to the time of host specialization. In most cases, these wild insects do not appear to have direct contact with humans and it remains to be determined whether these viruses possess heterogeneous infectivity and pathogenicity for humans. Nevertheless, concerns about the potential risk of virus spillover are increasing in accordance with the rapid expansion of human activity and abrupt ecological change (56–58). Unlike animal viruses, most plant viruses are transmitted to their host plants by vectors such as insects, fungi, and nematodes (18, 59). Many insects ingest viruses while feeding on the diseased plants (20, 60), and these viruses have developed the adaptive capacity to manipulate their insect vectors to facilitate their dispersion (18). The indispensable role of intermediate vectors in virus transmission and spread might explain the anomalous number of plant viruses observed in insects.

Analyses of these novel viruses have enabled a comprehensive and integrative reconstruction of the evolutionary history of insect viruses. There appear to be three major scenarios: (i) virus-host codivergence, which is typically characterized by a ladder-like topology in the phylogenies, representing viruses gradually coevolving with their hosts; (ii) virus speciation, in which the viral population undergoes evolutionary branching, resulting in distinct taxonomic groups (it is likely that both internal factors [e.g., genome rearrangement and segmentation] and external factors [e.g., physiological and ecological discrepancies in hosts] play important roles in virus speciation); and (iii) viral spillover, in which the original or ancestral form of the virus can be native or alien to host insects. The former may originate from the pervasive insect-specific viruses (ISV), and virus infection may become established in a new host following their introduction (54). In some circumstances, these viruses achieve productive and persistent infections in the new hosts independently of their original hosts (61, 62). Most of the insect-borne animal viruses, including some vertebrate-specific viruses, would likely fall into this category (43, 63). The prototype of the latter may be the invasive alien viruses introduced by the mechanics of feeding, as often employed in models of predation, herbivory, parasitism, and pollination. This is the choice for the spread of the majority of plant/fungus viruses (60).

In the present study, we have revealed complex viral communities and high levels of multiple infections in insects which may lead to community interactions with important biological consequences. A number of studies have examined the positive/negative effect of insect-specific viruses (ISV) on arbovirus replication in mosquitoes (54). These results highlight the importance of the identification and characterization of the hidden virus communities in trying to understand insect-associated viral diseases. However, great caution must be taken when characterizing a novel virus and its host range. As expected, the endogenous virus elements (EVEs) derived from RNA viruses are usually recognized in the gene pool of an insect genome (64, 65). We could not rule out the presence of EVEs in the present study, especially when the viral genome assembly is incomplete. The host distribution data could also be strongly influenced by unequal sample sizes. In our data set, fewer than 10 examples of RNA-Seq data were available for 20 insect orders, such as the fleas (*Siphonaptera*), representing a long-neglected ectoparasitic insect pest that entails high risk for zoonotic disease (66, 67). Here, extensive studies would provide more-detailed information about the evolution and ecology of virus infections in insects.

## MATERIALS AND METHODS

### Data set and virus discovery.

RNA-Seq data were retrieved from the NCBI Sequence Read Archive (SRA). The entire data set comprised 665 sets of RNA-seq data released by projects “1K Insect Transcriptome Evolution” (1KITE [http://www.1kite.org]) (68), “The evolution of Endopterygota” (69), “Ephemeroptera Phylogenomics” (70), and “Leptree II: non-ditrysia transcriptomes” (71) and other sequencing data for individual species (see Data Set S1 in the supplemental material). Each RNA-Seq data set was assembled *de novo* using the Trinity program with default parameters. The resulting contigs were first subjected to BLASTX searches against a curated database of the publicly available reference viral proteins with an E value cutoff of 1E−5; the database included all recognized RNA virus species approved by the International Committee on Taxonomy of Viruses (ICTV [https://talk.ictvonline.org/taxonomy/vmr/]). The putative viral contigs were then compared to the entire nonredundant protein database (nr) of NCBI using BLASTX to remove false positives, using the same cutoff E value. Viral contigs with unassembled overlaps were merged using the Seqman program implemented in the Lasergene software package (DNAStar). Among the putative viral contigs, those with predicted amino acid sequence lengths of >100 aa were retained in the following analysis. To facilitate the viral taxonomic classification, the putative viral contigs were also queried against a special database, with a small subset restricted to the conserved viral RNA-dependent RNA polymerase (RdRp) domain. To remove the false positives conferred by instrument carryover or cross-contamination, the initial reads in the sequencing library were mapped onto the viral contigs using Bowtie2 software with default parameters (72); highly homologous virus sequences (≥95% nt identity) with extremely low (<1%) mapping rates were considered to have been excluded in the subsequent analysis. Putative RNA viruses assigned a designation based on the corresponding hosts from which the virus-like sequences were identified and the virus categories to which they were assigned, followed by a numeral if two or more variants were identified within the same host.

### Virus genome annotation.

Contigs were annotated by the use of the RAST (Rapid Annotation using Subsystem Technology) server (73) and the online tool ORF Finder (http://www.bioinformatics.org/sms2/orf_find.html). Conserved domains within the predicted encoded proteins were identified using CD-search against the Conserved Domain Database (CDD) at NCBI. Sequence logos were drawn using the ggseqlogo package in R (74).

### Phylogenetic analysis.

Global phylogeny analyses were performed along with analyses of the reference viral genomes proposed by the International Committee on Taxonomy of Viruses (ICTV). To fully characterize the individual viral clades, the predicted amino acid sequences were also queried against the nonredundant protein database (nr) at NCBI. Viral protein sequences with significant E values were retrieved and used for phylogenetic reconstruction. In order to reduce computation time, we used the CD-HIT tool (75) to reduce sequence redundancy while maintaining sufficient virus diversity. Sequences were aligned by the use of MAFFT v7.273 and the L-INS-i algorithm (76) and were manually edited in Mega7 (77). Maximum likelihood phylogenetic trees were constructed with the best-fit substitution model implemented in IQ-TREE v1.6.5 (78). Statistical robustness and reliability were assessed using the ultrafast bootstrap (Ufboot) method with 1,000 replicates and the SH-like approximate likelihood ratio test (SH-aLRT) with 1,000 replicates. Finally, trees were visualized using FigTree v1.4.3 (available at http://tree.bio.ed.ac.uk/software/figtree/) and further edited using the online tool iTOL v4 (https://itol.embl.de/). Improvements in the phylogenies were achieved by removing divergent and ambiguous sequences.
